# Robotic versus laparoscopic right hemicolectomy with complete mesocolic excision: a retrospective multicenter study with propensity score matching

**DOI:** 10.3389/fonc.2023.1187476

**Published:** 2023-06-02

**Authors:** Yue Tian, Dehai Xiong, Ming Xu, Qi Fan, Huichao Zheng, Haode Shen, Bin Huang, Li Wang, Chunxue Li, Anping Zhang, Baohua Liu, Fan Li, Feng Gao, Weidong Tong

**Affiliations:** ^1^Department of General Surgery, Colorectal Division, Army Medical Center, Army Medical University, Chongqing, China; ^2^Department of Colorectum, Chongqing University Three Gorges Hospital, Chongqing, China; ^3^Department of Colorectum, The 940Hospital of Joint Logistics Support Force of Chinese People’s Liberation Army, Lanzhou, China

**Keywords:** right hemicolectomy, complete mesocolic excision, colonic cancer, laparoscopic surgery, robotic surgery

## Abstract

**Objective:**

During the past decade, the concept of complete mesocolic excision (CME) has been developed in an attempt to minimize recurrence for right-sided colon cancer. This study aims to compare outcomes of robotic versus laparoscopic right hemicolectomy with CME for right-sided colon cancer.

**Methods:**

We performed a retrospective multicenter propensity score matching study. From July 2016 to July 2021, 382 consecutive patients from different Chinese surgical departments were available for inclusion out of an initial cohort of 412, who underwent robotic or laparoscopic right hemicolectomy with CME. Data of all patients were retrospectively collected and reviewed. Of these, 149 cases were performed by a robotic approach, while the other 233 cases were done by laparoscopy. Propensity score matching was applied at a ratio of 1:1 to compare perioperative, pathologic, and oncologic outcomes between the robotic and the laparoscopic groups (*n* = 142).

**Results:**

Before propensity score matching, there were no statistical differences regarding the sex, history of abdominal surgery, body mass index (BMI), American Joint Committee on Cancer (AJCC) staging system, tumor location, and center between groups (*p* > 0.05), while a significant difference was observed regarding age (*p* = 0.029). After matching, two comparable groups of 142 cases were obtained with equivalent patient characteristics (*p* > 0.05). Blood loss, time to oral intake, return of bowel function, length of stay, and complications were not different between groups (*p* > 0.05). The robotic group showed a significantly lower conversion rate (0% *vs*. 4.2%, *p* = 0.03), but a longer operative time (200.9 min *vs*. 182.3 min, *p <* 0.001) and a higher total hospital cost (85,016 RMB *vs*. 58,266 RMB, *p <* 0.001) compared with the laparoscopic group. The number of harvested lymph nodes was comparable (20.4 *vs*. 20.5, *p* = 0.861). Incidence of complications, mortality, and pathologic outcomes were similar between groups (*p* > 0.05). The 2-year disease-free survival rates were 84.9% and 87.1% (*p* = 0.679), and the overall survival rates between groups were 83.8% and 80.7% (*p* = 0.943).

**Conclusion:**

Despite the limitations of a retrospective analysis, the outcomes of robotic right hemicolectomy with CME were comparable to the laparoscopic procedures with fewer conversions to open surgery. More clinical advantages of the robotic surgery system need to be further confirmed by well-conducted randomized clinical trials with large cohorts of patients.

## Introduction

In recent years, complete mesocolic excision (CME) was propagated, by Hohenberger et al. (1), in right-sided colon cancer following the established standard resection, which is total mesorectal excision (TME) for rectal cancer. It has been gradually accepted for its safety and efficacy in laparoscopy following some clinical studies (2, 3). In addition, several studies had shown that a lower rate of postoperative complications was observed in laparoscopic right hemicolectomy for right-sided colon cancer, and similar results in disease-free survival (DFS) and overall survival (OS) were reported by comparison with those in open right hemicolectomy (4–6).

Following the principle of CME, the central vascular ligation with complete exposure and lymphadenectomy along the superior mesenteric axis may potentially increase the technical difficulties of minimally invasive surgery, especially in right colon cancer (1). Lately, robotic surgery has increasingly penetrated in the practice of general surgery. Theoretically, the robotic characteristics, including naked eye three-dimensional (3D) vision, ergonomic design, simulated wrist surgical instrument, and tremor filtration, may conduct a more precise and fine dissection (7). It is generally believed that robotic systems are absolutely advantageous to perform operations within narrow spaces, such as rectal cancer and prostate surgery (8). However, the reported outcomes regarding the significant advantages of robot-assisted colectomy are debatable and conflicting, and, to date, the feasibility, safety, and efficacy of the robotic surgery in right hemicolectomy are still controversial (9–12). Thus, using propensity score matching analysis to avoid the bias in patient selection, the aim of our multicenter study is to evaluate the outcomes of robotic versus laparoscopic right hemicolectomy with CME for right colon cancer.

## Materials and methods

### Patients

**Figure 1:**
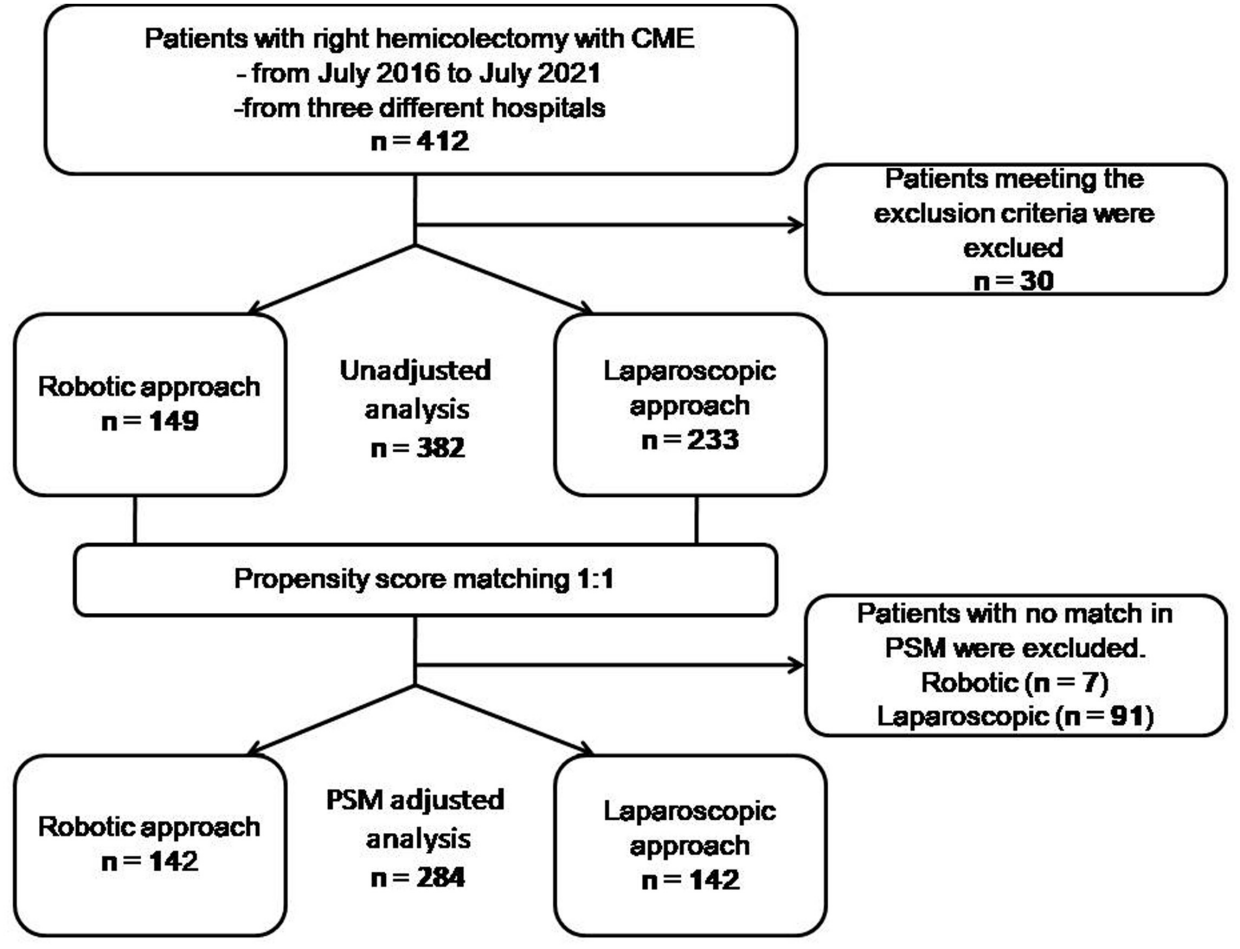
Flow diagram of included and excluded participants and the statistical analyses performed. *PSM* propensity score matching.

The present study was approved by the Ethics Committee, and informed consent was obtained from all patients before the operation. All consecutive patients who underwent robotic or laparoscopic right hemicolectomy with CME from July 2016 to July 2021 at three Chinese surgical departments (Department of General Surgery, Army Medical Center, Chongqing; Department of Colorectum, Chongqing University Three Gorges Hospital, Chongqing; and Department of Colorectum, the 940th Hospital of Joint Logistics Support Force of Chinese People’s Liberation Army, Lanzhou) were included in the study. The study was registered with http://www.ClinicalTrials.gov (NCT05457426). A retrospective review of multicenter institutional database was conducted. The Da Vinci Si Surgical System (Intuitive Surgical, Sunnyvale, CA, USA) has been employed since 2016 in the three centers and one surgeon in each hospital participated in the study. From July 2016 to July 2021, an initial cohort of 412 consecutive patients underwent robotic or laparoscopic right hemicolectomy with CME in three hospitals (**Figure 1**). The exclusion criteria included a patient for any indications such as multiple primary colorectal tumors, peritoneal metastasis, pelvic or distant organs, presence of bowel obstruction or perforation, neuroendocrine tumors, lymphomas, and other malignant tumors. With 30 cases meeting the exclusion criteria, 382 cases, including 204 male and 178 female patients, were available for inclusion. Of these, 149 cases who were subjected to the robotic approach were classified as the robotic group, while the other 233 cases who underwent laparoscopy were classified as the laparoscopic group. There were no selection criteria for the robotic and laparoscopic approaches. Choice of surgical approach was based on the availability of the robotic system. All robotic and laparoscopic operations were performed by the surgeons who had an experience of more than 150 prior laparoscopic right hemicolectomies.

### Surgical technique

**Figure 2:**
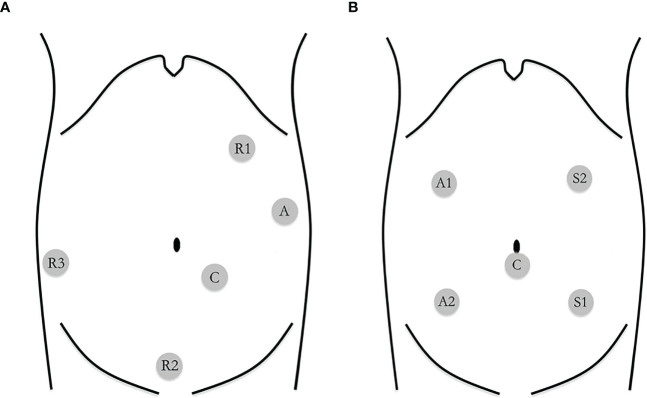
**(A)** Trocar placement in robotic-assisted right hemicolectomy R1: robotic arm 1; R2: robotic arm 2; R3: robotic arm 3; C: camera; A: assistant. **(B)** Trocar placement in laparoscopic-assisted right hemicolectomy S1: surgeon's left hand; S2: surgeon's right hand; A 1: assistant's left hand; A2: assistant's right hand; C: camera.

Bowel preparation was conducted by polyethylene glycol for all patients 1 day before surgery. The patient was placed on the left tilted operating table (to allow the small intestine to fall away from the point of interest), with arms along the body and legs closed. The distribution of trocars was placed according to the position of Intuitive Surgical Inc. for robotic colectomy (13). The robot was set to come and dock from the right shoulder of the patient. Three robotic 8-mm trocars (R1, R2, and R3) and two 12-mm trocars (camera and assistant port) were used for the robotic procedure (**Figure 2**). One working arm carrying a monopolar cautery hook/scissors for dissection was located in the left upper quadrant port (R1). The other two working arms carried bipolar forceps in the suprapubic port (R2), and Cadiere’s fenestrated forceps in the right lower quadrant port (R3) that was used to keep the superior mesenteric axis in traction. After gentle cephalad traction on the transverse mesocolon with the grasp in R3, the assistant grasped the ileocecal valve through the assistant port to put the ileocolic vascular pedicle on tension and the ileocolic vessels were identified and lifted up with R2. The posterior peritoneum was then opened just below their prominence and along the left side of the anterior aspect of the superior mesenteric vein. The ileocecal artery and vein were ligated with clips. Then, the right mesocolon was mobilized from the Gerota’s fascia. The duodenum and the right ureter were identified and protected. Right colic vessels (if present), middle colic veins and the right branch of the middle colic artery could be easily and safely ligated with clips at their roots along the right border of the superior mesenteric axis and at the Henle sinus. A CME was performed by sharp dissection of the posterior visceral fascial layer from the parietal one along Gerota’s and Fredet’s fascias, thus exposing the duodenum and the pancreatic head and providing a specimen with intact visceral fascial layers on both sides. After that, we mobilized the transverse colon by dividing the gastrocolic ligament from the level of the middle colic artery and towards the hepatic flexure (medial to lateral mobilization). Then, the ascending colon was mobilized by dividing the peritoneal attachments in the white line of Toldt across the right paracolic gutter. After complete colonic detachment, the R3 port site was expanded to a 4-cm muscle-splitting incision and the free bowel was extracted through a wound protector. Then, the transverse colon and ileum (10 cm away from the ileocecal valve) are divided with the use of linear staplers. A side-to-side isoperistaltic mechanical anastomosis (with a double-layered continuous suture closing the enterotomies carried out for the introduction of the 60-mm linear stapler) was performed. A drainage tube was left through the robotic trocar incision (R3) and placed in the right paracolic gutter.

Laparoscopic right hemicolectomy was carried out following the same surgical principles. We used two 12-mm trocars (left upper quadrant and umbilicus) and three 5-mm trocars (left lower quadrant, right upper, and lower quadrant) (**Figure 2**). The procedure followed the same key surgical steps of the robotic approach, including CME. An ultrasonic device was used for dissection. The specimen was extracted *via* a right-side transrectus incision.

Patients were treated postoperatively with adjuvant chemotherapy when indicated and were followed up at 3-month intervals for the first 2 years and 6-month intervals thereafter.

### Measurements

Patients’ clinical characteristics included sex, age, history of abdominal surgery, body mass index (BMI), American Joint Committee on Cancer (AJCC) staging system, tumor location, and center. Perioperative, pathological, and oncological outcomes included conversion rates (percent), operative time (skin to skin, minutes), estimated blood loss (ml), oral intake (days), return of bowel function (days), length of stay (days), total hospitalization cost (RMB), intraoperative and postoperative complications (*N*, %) (according to the Clavien–Dindo classification scale) (14), number of harvest lymph nodes (*N*), number of patients with lymph node metastasis (*N*, %), tumor differentiation (*N*), positive margins (*N*, %), DFS (months), and OS (months). Disease recurrence was defined as a positive result on the CT or ultrasound test or elevated oncologic markers in the blood samples (i.e., CEA, CA125, and CA 19-9).

### Statistical analysis

All statistical analyses were performed using SPSS version 23.0 (IBM, Endicott, New York, USA). To reduce the bias due to the non-randomization nature of patient selection, we conducted a propensity score matching model. A logistic regression model was performed to estimate the propensity score of each patient using the covariates including sex, age, history of abdominal surgery, BMI, AJCC staging system, tumor location, and center. A nearest neighboring matching method was used to identify the best robotic patient for each individual laparoscopic patient. The propensity scores were used to match two groups of patients with a ratio of 1:1 and comparisons of the variables were performed before and after propensity score matching.

Descriptive statistics were presented as mean ± standard deviations and ranges for numeric variables and as proportions for categorical variables. Categorical variables were presented as number and percentage. Pearson’s chi-squared test or Fisher’s exact test were employed for categorical variables, as appropriate, while Student’s *t*-test was used for continuous variables. These analyses were performed for both groups. DFS and OS rates were analyzed using Kaplan–Meier survival analysis and compared between both groups using the log rank test. *p* < 0.05 was considered statistically significant.

## Results

### Baseline characteristics

We retrieved data on a total of 412 patients operated in the given period (**Figure 1**). The comparison of demographic data before propensity score matching is compiled in **Table 1**. A significant difference was observed in age between groups (*p* = 0.029). All of the variables, regarding sex, age, history of abdominal surgery, BMI, AJCC staging system, tumor location, and center, were well-balanced and showed no significant differences (*p* > 0.05) after applying propensity score matching at a ratio of 1:1 for both the robotic (*n* = 142) and laparoscopic groups (*n* = 142).

**Table 1:** Baseline characteristics of patients.

Characteristics	Overall cohort				After propensity score-matching			
	Robotic group	Laparoscopic group	t/χ^2^	*P*	Robotic group	Laparoscopic group	t/χ^2^	*P*
	n = 149	n = 233			n = 142	n = 142		
Sex (male/female, N)	81/68	123/110	0.09	0.764	74/68	79/63	0.354	0.552^b^
Age (years)	64.1(11.2)	61.4(11.8)	2.197	0.029*	63.2(10.8)	63.4(11.3)	0.172	0.864^a^
BMI (kg/m^2^)	22.5(2.4)	22.3(2.7)	0.627	0.531	22.5(2.4)	22.5(2.7)	0.031	0.975^a^
History of abdominal surgery	34	49	0.171	0.679	11	9	0.215	0.643^b^
AJCC staging (n)			4.445	0.108			3.168	0.205^b^
I	24	23			21	12		
II	68	127			66	76		
III	57	83			55	54		
Tumor location			1.381	0.71			5.684	0.128^b^
Ileocecal region	18	22			4	2		
Ascending colon	67	115			21	29		
Hepatic flexure	33	54			13	5		
Transverse colon	31	42			10	12		
Centers			1.733	0.42			0.465	0.793^b^
CenterA	61	108			61	62		
CenterB	23	39			23	19		
Center C	65	86			58	61		

Values are expressed as mean (SD = standard deviation) or n (%)

Robotic group: robot-assisted right hemicolectomy with CME; Laparoscopic group: laparoscopic right hemicolectomy with CME;

BMI: body mass index; AJCC: American Joint Committee on Cancer

a Student’s t test; Pearson’s Chi squared test

*P< 0.05 was considered statistically significant.

### Perioperative outcomes

After matching, perioperative outcomes are listed in **Table 2**. Blood loss, time to oral re-intake, return of bowel function, and length of stay were similar between both groups (*p* > 0.05). Operative time was significantly longer in the robotic group (200.9 min *vs*. 182.3 min, *p <* 0.001), but a statistically significant difference was found in conversion rates between the two groups (robotic group 0% *vs*. laparoscopic group 6/142, 4.2%; *p* = 0.03). Laparoscopic conversions were due to severe adhesions in four patients, and massive bleeding (superior mesenteric vein injury and the middle colon artery injury) in two patients. No intraoperative complication occurred in the robotic series.

**Table 2:** Comparison of perioperative and pathologic outcomes between the robotic and laparoscopic groups.

	Robotic group (*n* = 142)	Laparoscopic group (*n* = 142)	*t*/*χ^2^*	*p*
Conversion rates [*N* (%)]	0 (0%)	6 (4.2%)	–	0.030^b*^
Operative time (min)	200.9 (62.1)	182.3 (33.4)	3.676	<0.001^a*^
Blood loss (ml)	76.8 (48.3)	75.6 (65.1)	0.186	0.852^a^
Oral re-intake (days)	2.9 (2.3)	3.3 (2.7)	0.802	0.425 ^a^
Return of bowel function (days)	3.0 (1.0)	2.8 (0.9)	0.924	0.358^a^
Length of stay (days)	8.7 (3.4)	9.0 (4.7)	0.537	0.592^a^
Total hospitalization cost (RMB)	85,016 (14,920)	59,266 (13,692)	15.152	0.000^a*^
Mortality [*N* (%)]	0 (0%)	0 (0%)	–	1.000^b^
Intraoperative complications	0 (0%)	2 (1.4%)	2.014	0.156^b^
Superior mesenteric vein injury	0 (0%)	1		
The middle colon artery injury	0 (0%)	1		
Overall postoperative complications within 30 days after surgery [*N* (%)]	22 (15.5%)	23 (16.2%)	0.026	0.871^b^
(Clavien–Dindo grades I and II)	5	5		
Wound infection	5	5		
Inflammatory ileus	1	3		
Gastroparesis	2	3		
Respiratory complications	2	0		
Ulcer bleeding	1	0		
Lymphatic leakage	1	1		
Acute urinary retention	0	1		
Acute heart failure	1	2		
Intra-abdominal abscess	0	1		
Anastomotic bleeding				
(Clavien–Dindo grades III and IV)	1	0		
Pulmonary embolism	1	2		
Hemoperitoneum	1	0		
Acute respiratory failure	20.4(3.8)	20.5(6.1)	0.175	0.861^a^
Harvested lymph nodes (*N*)	55(38.7%)	54(38.0%)	0.015	0.903^b^
Number of patients with lymph node				
metastasis [*N* (%)]			3.650	0.302^b^
Differentiation, *N*	4	6		
Well	108	98		
Moderate	16	14		
Poor	14	24		
Mucinous carcinoma	0	0	–	1.000^b^
Positive margins [*N* (%)]				

Values are expressed as mean (SD = standard deviation) or n (%).

a Student’s t-test;

b Pearson’s Chi-squared test or Fisher’s exact test.

*p < 0.05 was considered statistically significant.

Grading of postoperative complications was based on the Clavien–Dindo classification system.

Overall postoperative complications within 30 days are reported in **Table 2** and similar between the groups (robotic group 15.5% *vs*. laparoscopic group 16.2%; *p* > 0.05). Two patients in the laparoscopic group suffered from massive hemoperitoneum from an epiploic vessel, while one patient in the robotic group did from the middle colic artery due to clip displacement and so required reoperation on postoperative day 1. There is one patient who suffered from intra-abdominal abscesses. The patient had lower abdominal pain and diarrhea. The complaint was diagnosed by abdominal CT and was cured by ultrasound-guided abdominal puncture and drainage. No death or anastomotic leakage occurred in both groups. Higher total hospitalization cost was observed in the robotic group (85,016 RMB *vs*. 58,266 RMB, *p <* 0.001).

### Pathological outcomes

Pathological outcomes are reported in **Table 2** and comparable between both groups (*p* > 0.05). The mean number of harvested lymph nodes was 20.4 versus 20.5 in the robotic and laparoscopic groups, respectively (*p* > 0.05). Lymph node metastasis was seen in 55 (38.7%) cases in the robotic group and 54 cases (38.0%) in the laparoscopic group. No residual cancer cell at the resection margin was found in both groups.

### Oncologic outcomes

**Figure 3:**
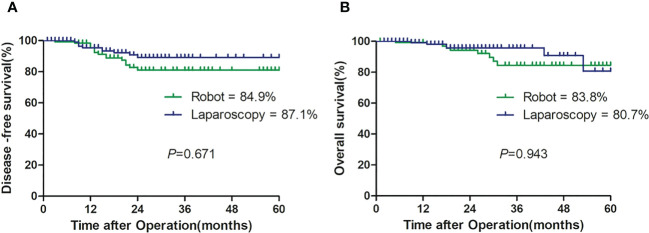
Kaplan - Meier survival curve showing disease-free survival **(A)** and overall survival **(B)**.

All patients in both groups were successfully followed up with a median follow-up of 19 months (range, 1–60). The DFS rates and OS rates were 84.9% versus 87.1% (*p* = 0.841) and 83.8% versus 80.7% (*p* = 0.310) in the robotic and laparoscopic groups, respectively (**Table 3**; **Figure 3**).

**Table 3:** DFS and OS between the robotic and laparoscopic groups.

	Robotic group (*n* = 142)	Laparoscopic group (*n* = 142)	*t*/*χ^2^*	*p*
Median follow-up (months)	18.5 (1–60)	20 (1–60)	1.030	0.300^a^
Disease-free survival (%)	84.9%	87.1%	0.040	0.841^b^
Overall survival (%)	83.8%	80.7%	1.030	0.310 ^b^

Values are expressed as median (ranges) or n (%).

a Student’s t-test;

b Kaplan–Meier survival analysis.

## Discussion

There have been a number of studies looking at the feasibility, safety, and long-term outcomes of laparoscopic CME (2, 15–18). If compared with left hemicolectomy, dissection of the vascular pedicle is considered to be the most complex step of laparoscopic right hemicolectomy with CME (13). Following the concept of CME, the level of vessel ligation and central lymphadenectomy with complete exposure of the superior mesenteric vein and pancreatic head can be difficult steps during minimally invasive right hemicolectomy (19).

Since the mid-2000s, robotic surgery has been performed in some institutions as a helpful alternative to a conventional laparoscopic surgery in a variety of colorectal procedures with the technological advantages, which may overcome the limitation of straight laparoscopic instruments (20). Robotic technology has been widely used in the treatment of rectal cancer with favorable outcomes (21, 22). Theoretically, the technical features of the three-dimensional and magnified vision may make the surgical anatomy so clear that it is more conducive to dissection of the vascular pedicle and central lymphadenectomy for CME (13). However, to date, data on potential advantages of robotic versus laparoscopic right hemicolectomy are still lacking, and only a few comparative studies with short-term outcomes exist between robotic and laparoscopic right hemicolectomy (19). A comprehensive literature review by Ahmad et al. demonstrated that robot-assisted surgery had greater advantages in narrow spaces, such as rectal cancer surgery, while no obvious advantage in colonic cancer surgery (23). A systematic review and meta-analysis by Solaini et al. suggested that robotic surgery represented lower rates of conversion, faster recovery of bowel function, shorter length of stay and lower rates of complication (24). It was undeniable that longer operative time and higher cost were observed in robotic surgery compared with those in laparoscopic surgery (25).

In this study, we demonstrated the safety and efficiency of both robotic and laparoscopic right hemicolectomy in three institutions with no significant difference observed in blood loss, time to oral re-intake, return of bowel function, length of stay, complications, and pathological and oncological outcomes. We reported a longer operative time in the robotic group compared with laparoscopy (200.9 min *vs*. 182.3 min, *p <* 0.001), which was not surprising considering prior publication (26). The operative time in our study was shorter than that in the studies by Spinolio et al. (7) (101 cases, 279 min) and Hannan et al. (27) (35 cases, 216 min). Although each surgeon had an experience of more than 1,000 prior laparoscopic resection of colorectal cancer, robotic surgery in our institutions had only developed for several years. It was possible that this was due to the robotic learning curve, and the operative time could be shortened with an accumulation of experience (28). In my personal opinion, the time of dissection and mobilization in robotic surgery might be comparable to that in laparoscopic surgery.

Previous surveys reported conversion rates for laparoscopic colonic resections ranging from 6.9% to 15.6% (7). In our study, a reduction conversion rate (0% *vs*. 4.2%, *p* = 0.03) was observed in favor of the robotic group. The lack of conversions in the robotic group is consistent with the majority of robotic right hemicolectomy papers (24). Reducing conversions has meaningful impact on patient outcomes. Owing to the high morbidity and prolonged length of hospital stay for patients, keeping the incidence of conversions to laparotomy as low as possible is crucial. Although many factors that are heterogeneous and difficult to control contribute to a conversion, a consistent and noteworthy decrease is reported in conversion associated with the robotic approach compared to laparoscopy, with a 58.5% relative reduction in conversion events (29). It is generally accepted that conversion should not be considered as a complication or a failure at an individual case level. However, in general, conversion rates have been gradually agreed as a measure of quality. Emergency conversions due to a catastrophic event such as massive bleeding and elective conversions due to failure to progress during the procedure can broadly be regarded as the reasons for a conversion. In our study, two patients with massive bleeding (superior mesenteric vein injury and the middle colon artery injury) occurred in laparoscopic conversions and none was observed in the robotic group. This may be due to the limitations of the surgeon on the left side of the patient and the straight laparoscopic instruments, while the rotating mechanical arms of the robot may play an important role during the vascular dissection. The emerging data suggest that the robotic approach is generally associated with a reduction in the elective conversions (30). The reason for the significant decrease in conversion with the robotic approach could be a reflection of some technological advantages of the robotic platform compared to the laparoscopic approach during more difficult dissection. This study cannot determine the causality, but these technological advantages could contribute to the observed differences.

A major drawback of robotic surgery was the great expenses due to operation room charge. Multiple studies showed the cost inefficiency of robotic surgery in the treatment of colorectal cancer, which limited the penetrance of robotic surgery (24). In our trial, it could not be ignored that the hospitalization cost in the robotic group was noticeably higher than that in the laparoscopic group. Therefore, we look forward to further development in robotics leading to the reduction of costs and improvement of outcomes, as occurred in the past for laparoscopic surgery (25).

Thirty-day major complication rates in laparoscopic and robotic series were low and comparable between both groups (robotic group 15.5% *vs*. laparoscopic group 16.2%; *p* > 0.05), suggesting that robotic and laparoscopic surgery were feasible and safe. This was lower than previous studies by Yozgatli et al. (31) (29% *vs*. 25%) and by Lujan et al. (32) (31.8% *vs*. 28.0%).

In a recent study, robotic right hemicolectomy with CME was associated with a better number of lymph nodes than laparoscopic surgery, suggesting that robotic surgery might perform a better CME (33). It was reported that the incidence of central mesocolic lymph node metastases for right-side cancers varied between 1% and 22% (34). Our results showed no significance in the number of harvested lymph nodes between both groups (20.4 versus 20.5, *p* > 0.05). Although the three-dimensional and magnified view in the robotic surgery system might be beneficial to CME and D3 lymphadenectomy, no significant difference should be observed theoretically in the number of lymph node dissection by the same surgeon between both approaches.

In addition, the pathologic stage was one of the most important factors to determine prognosis that was included for propensity score matching. Although there was no significant difference in the pathologic stage between both groups (*p* = 0.108), the groups were well balanced after matching (*p* = 0.205). Based on the results, we found no significant difference in the estimated 2-year DFS (84.9% *vs*. 87.1%, *p* > 0.05) and OS (83.8% *vs* 80.7%, *p* > 0.05) (**Table 3**). There were only a few reports on oncologic outcomes of robot-assisted hemicolectomy with CME. It was reported by Kang et al. (35) that the 5-year DFS from the follow-up results of 96 patients undergoing right hemicolectomy, including 33 cases of open surgery, 43 cases of laparoscopic surgery, and 20 cases of robot-assisted surgery, was 87.7%, 84.0%, and 89.5%, respectively (*p* = 0.830), and the 5-year OS among the three groups was 86.4%, 79.2%, and 73.1%, respectively (*p* = 0.916). Moreover, Park et al. (9) reported that the 5-year DFS and OS with 71 patients undergoing right hemicolectomy including 36 cases of laparoscopic surgery and 35 cases of robot-assisted surgery were not different between robotic and laparoscopic groups (77.4% *vs*. 83.6%, *p* = 0.442 and 91.1% *vs*. 91.0%, *p* = 0.678, respectively). Similar to the above studies, our study had favorable 2-year oncologic outcomes.

Our study had some limitations. Firstly, due to its retrospective nature, selection bias would be inevitable despite propensity score matching. Secondly, although our multicenter study involves three high surgical volumes, much bigger randomized controlled studies are necessary in the future. Thirdly, the BMI of Asian patients is lower than that of Western patients, which may limit the generalizability in our study. Fourthly, the bias of the learning curve of the robotic surgeons cannot be ignored.

In conclusion, our findings suggested that robot-assisted right hemicolectomy with CME by experienced surgeons was as safe and feasible as conventional laparoscopy with excellent outcomes, despite the longer operative time and higher hospitalization cost of robotic surgery. Robotic surgery, even within the learning curve, allows for the completion of technically difficult surgical procedures without conversion. Further studies with randomized controlled trials and long follow-up are needed before recommending CME in routine practice, and studies after the robotic learning curve may be needed to better confirm the advantages of robotic surgery.

## Data availability statement

The raw data supporting the conclusions of this article will be made available by the authors, without undue reservation.

## Ethics statement

The trial has been approved by the Medical Ethics Committee of Army Medical Center (reference number 2021-248). The patients/participants provided their written informed consent to participate in this study. Written informed consent was obtained from the individual(s) for the publication of any potentially identifiable images or data included in this article.

## Author contributions

YT Data curation-Equal, Formal analysis-Equal, Writing – original draft-Equal. FG Resources-Equal, Writing – original draft-Equal. MX Investigation-Equal, Resources-Supporting. QF Investigation-Equal, Resources-Supporting. HZ Software-Equal. HS Methodology-Equal. BH Formal analysis-Equal, Investigation-Equal. LW Formal analysis-Equal, Investigation-Equal. CL Formal analysis-Equal. AZ Formal analysis-Equal. BL Conceptualization-Equal. FL Resources-Equal, Supervision-Equal, Writing – review and editing-Supporting. DX Resources-Equal, Supervision-Equal, Writing – review AND editing-Supporting. WT Funding acquisition-Equal, Project administration-Equal, Resources-Equal, Supervision-Equal, Writing – review and editing-Equal. All authors contributed to the article and approved the submitted version.
